# Early presentation of HIV-1 KF11Gag and KK10Gag protective epitopes facilitate rapid CD8+ T cell activation and killing of virus infected cells

**DOI:** 10.1186/1742-4690-9-S2-P255

**Published:** 2012-09-13

**Authors:** HN Kløverpris, R Payne, JB Sacha, F Chen, J Rasaiyaah, G Towers, P Goulder, JG Prado

**Affiliations:** 1 University of Oxford, Oxford, UK; 2 Oregon Health & Science University, Portland, OR, USA; 3 Royal Berkshire Hospital, Reading, UK; 4 University College London, London, UK; 5 AIDS Research Institute IrsiCaixa, Barcelona, Spain

## Background

CD8+ T cells are major players for the antiviral immunity against HIV-1 through recognition of viral epitopes presented on the surface of infected cells. However, the kinetics and timing of HIV-1 epitope presentation remains poorly understood but nonetheless crucial for development of a successful CD8+ T cell based vaccine.

## Methods

Epitope presentation and killing of virus infected cells was assessed in HIV-1 susceptible T cell lines, H9, U937, primary CD4+, and B cell lines expressing HLA-B*5701 or B*2705 by synchronized infection with HIV-1 or VSV-HIV-1 to determined the contribution of incoming particles to epitope presentation. Infected cells were co-cultured with CD8+ T cell lines specific for the epitopes KF11Gag, KK10Gag, KY9Pol and VL9Vpr. Kinetics of epitope presentation were monitored by the production of CD107b and MiP1ß in the co-culture at 0, 3, 6, 18 and 24 hours post-infection. In addition, killing of infected cells was determined in paralleled by the decrease of CD4+/p24 Gag+ cells.

## Results

We comprehensively studied the kinetics of antigen presentation of the KF11Gag and KK10Gag epitopes, restricted by protective HLA alleles B*5701 and B*2705, and compared these to KY9Pol and VL9Vpr epitopes, in a single cycle of virus replication. We observed differences in epitope presentation kinetics with early presentation within 3 hours post-infection, for KF11Gag, KK10Gag and KY9Pol epitopes, but only late presentation for VL9Vpr. In addition, we illustrate how early presentation relies on antigen processing from incoming virus, which correlates with rapid CD8+ T cell activation and clearance of virus-infected cells.

## Conclusion

Our data strongly support the importance of identifying early-presented HIV-1 epitopes to eliminate infected cells before the release of new infectious viral particles.

